# CTCF in parvalbumin-expressing neurons regulates motor, anxiety and social behavior and neuronal identity

**DOI:** 10.1186/s13041-022-00916-9

**Published:** 2022-04-04

**Authors:** Liron Davis, Prudhvi Raj Rayi, Dmitriy Getselter, Hanoch Kaphzan, Evan Elliott

**Affiliations:** 1 Bar Ilan University, Azrieli Faculty of Medicine, Hanrietta Sold 8, 13215 Safed, Israel; 2 Sagol Department of Neurobiology, University of Haifa, Haifa, Israel

**Keywords:** CTCF, Anxiety, Autism, Neurodevelopment, Parvalbumin, Inhibitory neuron

## Abstract

CCCTC-binding factor (CTCF) is a regulator of chromatin organization and has direct effects on gene transcription. Mutations in CTCF have been identified in individuals with neurodevelopmental conditions. There are wide range of behaviors associated with these mutations, including intellectual disabilities, changes in temperament, and autism. Previous mice-model studies have identified roles for CTCF in excitatory neurons in specific behaviors, particularly in regards to learning and memory. However, the role of CTCF in inhibitory neurons is less well defined. In the current study, specific knockout of CTCF in parvalbumin-expressing neurons, a subset of inhibitory neurons, induced a specific behavioral phenotype, including locomotor abnormalities, anxiolytic behavior, and a decrease in social behavior. The anxiolytic and social abnormalities are detected before the onset of locomotor abnormalities. Immunohistochemical analysis revealed a disbalance in parvalbumin-expressing and somatostatin-expressing cells in these mice. Single nuclei RNA sequencing identified changes in gene expression in parvalbumin-expressing neurons that are specific to inhibitory neuronal identity and function. Electrophysiology analysis revealed an enhanced inhibitory tone in the hippocampal pyramidal neurons in knockout mice. These findings indicate that CTCF in parvalbumin-expressing neurons has a significant role in the overall phenotype of CTCF-associated neurodevelopmental deficits.

**Supplementary Information:**

The online version contains supplementary material available at 10.1186/s13041-022-00916-9.

## Introduction

The human genome is highly complex and dynamically packaged into several levels of organization, from chromatin fibers and nucleosomes to chromosomal domains that occupy a specific territory in the nucleus [1]. Chromosome compaction is partly achieved by formation of chromosome loops [2]. CCCTC-binding factor (CTCF), a11-zinc finger protein, has an essential role in chromatin architecture[3–5]. The binding factor CTCF can link chromatin domains through long range interactions between genomic regions, by packaging promotor-enhancer/ promotor- repressor, and therefore controlling gene expression and chromatin organization.

Recent studies show that CTCF is involved in neurological diseases and human cognition [6]. Individuals with de novo mutations in CTCF gene, which leads to haploinsufficiency for CTCF*,* have an intellectual disabilities, autistic features, microcephaly, and growth retardation [7–10]. Although the genetic mutations were located on different position in the gene encoding CTCF (N-terminal region/ C-terminal region/ Zinc finger domain), the clinical features were similar. A separate genetic study determined an association between Single-nucleotide polymorphism (SNPs) in the genomic vicinity of CTCF and schizophrenia (SCZ) [11]. The study detected strong association between SNPs in the genes CTCF and CACNB2 and schizophrenia, using a gene pathway-based approach.

Not only mutations in CTCF affect brain development and function, rather modifications in CTCF binding sites have also been implicated in neuropsychiatric phenotype [12–14]. For instance, CTCF directly binds to the promoter of FMR1 gene, the causative gene in Fragile x syndrome, and regulate its transcription [15]. In addition, mutations in genes encoding proteins that complex with CTCF such as SMC3, MECP2 and CHD8 are also known to be involved in neurodevelopmental disorders, showing the importance of CTCF in function and development of the central nervous system [16–19].

Full body knock-out (KO) of CTCF in both alleles causes lethality. Therefore, conditional knockout (cKO) mice are essential to determine the role of CTCF in neurons [20]. CTCF knockdown in neuroprogenitor cells leads to changes in neuroprogenitor cell proliferation and differentiation in early cortex formation and death upon birth [21]. Two separate studies found that CTCF knockout specifically in excitatory neurons induces synapse and dendrite structural abnormalities, expression of clustered protocadherins (Pcdh), and early lethality [21–24]. In addition, CTCF ablation in camkiia-expressing neurons decreases learning capabilities, social behaviors, experience-dependent gene expression and induces abnormal microglia and enhanced inflammation-related gene Transcription [24, 25]. These studies further demonstrate the role of CTCF in behavior.

In a study of the role of CTCF in differentiation of GABAergic interneurons, CTCF deletion in the MGE derived cortical interneurons leads to changes in cell specification and migration. These finding demonstrate a role for CTCF in cell fate determination during differentiation [26, 27]. However, little is known about the function of CTCF in adult mice and the role of CTCF in different neuronal populations after establishment of cell identity. In this study we investigate the role of CTCF in a specific subtype of GABAergic interneurons by crossing mice carrying a loxP allele of CTCF [28] with mice transgenic for PV-Cre, which express Cre recombinase in inhibitory neurons that express parvalbumin (PV). Mice with deletion of CTCF in parvalbumin-expressing neurons induced a decrease in anxiety-like behavior and a social impairment at early age, followed by gradual deficits in motor function. CTCF cKO mice displayed a depletion in parvalbumin + neurons and increase in somatostatin + neurons in the hippocampus and cortex while no cell apoptosis was seen. Single nuclei RNA sequencing and electrophysiology analysis determined an upregulation of voltage gated ion channel activity and strengthening of inhibitory neuron identity in parvalbumin-expressing neurons. These results show that CTCF regulates behavior and motor function in inhibitory neurons at developmental time points after neuronal differentiation.

## Materials and methods

### Mice

Mice were housed in a temperature-controlled barrier facility maintained at 24 °C in a 12-h light/dark cycle. Mice had access to fresh food and water ad libitum. To generate CTCF cKO specifically in parvalbumin inhibitory neurons, PV-Cre line was crossed to ${CTCF}^{flox/flox}$ mice. The double transgenic ${PV-Cre}^{+/-}$, ${CTCF}^{flox/+}$ were backcrossed to ${CTCF}^{flox/flox}$ mice to produce ${PV-Cre}^{+/-}$,${CTCF}^{flox/flox}$ (cKO) and ${PV-Cre}^{-/-}$,${CTCF}^{flox/flox}$ (WT) mice. These two lines were crossed in all experiments. Therefore, all offspring were ${CTCF}^{flox/flox}$, and half were with ${PV-Cre}^{+/-}$. The mice without ${PV-Cre}^{+/-}$ were the control animals. All experimental protocols were approved by the Animal Care and Use Committee of faculty of medicine, Bar- Ilan University, Israel.

### Behavioral testing

Mice were habituated to the behavioral room for at least 1 h before commencement of each test. Each test was performed on a separate day, with one day rest between each test. A camera filmed the movement, and the Noldus Software “EthoVision” was used to track the behavior of the animals.

### Rotarod test

The test was carried out with an accelerating Rotarod (Med Associates, St. Albins, VT). The speed of the Rotarod was set to 40 r.p.m. The amount of time each mouse spent on the rod was measured. The latency to fall was recorded three times with a 300 s cutoff time and average was calculated between the three independent trials.

### Open field test

Anxiety-like and locomotor behavior was determined in the open field. The size of the box is 50 × 50 cm. The mouse is placed in a corner of the box and is allowed to explore for 10 min. We recorded, using the EthoVision XT 10/11 software (Noldus), the distance moved and time spent in the entire box and in center square (25 × 25 cm). Testing was performed under light of 120 lx.

### Limb clasping reflex

The mouse is lifted by their tail 30 cm above the floor for 10 s. Individual measures are scored on a scale of 0–3 [29]. If the hindlimbs are consistently splayed outward, it is a score of 0. If one hindlimb is retracted toward the abdomen, it receives a score of 1. If both hindlimbs are partially retracted toward the abdomen, it receives a score of 2. If its hindlimbs are entirely retracted and touching the abdomen, it receives a score of 3. Each test is performed three times to ensure that the score is reproducible.

### Light/dark box test

The mouse is placed in a dark plastic chamber (75 × 75 cm) with an opening to highly lit chamber (~ 1200 LUX). The mouse is free to move between the two chambers for 5 min. During this time, a camera films and tracks the behavior of the animals, including where they are found inside the box, velocity, distance traveled, etc.

### Elevated plus maze

The mouse is placed in the center of a four arms maze. Each arm is 30 cm in length, two are closed and two are open. The maze is one meter high. The mouse is free to choose which arm it enters for a 5 min period. During this time, a camera films and tracks the behavior of the animals, including where they are found in the maze, velocity, distance traveled, etc.

### Contextual and cued fear conditioning

First, on the training day each mouse was habituated to the fear conditioning cage for 5 min. Each mouse was placed into the conditioning chamber (10.5 × 10.5 × 10.5 cm) and allowed to explore freely for 2 min and a tone (75 dB) was sounded as the conditioned stimulus for 30 s followed by a two second mild foot-shock (0.7 mA) as the unconditioned stimulus. After a 1-min break, another tone and shock was administered and then the mouse was returned to the home cage 1 min after the second tone-shock pair. The next day, the mice were placed back into the conditioning chamber for 5 min and their freezing behavior was measured during this time period as a measure of contextual memory. Three hours after context testing, the mice were placed into a different chamber with a novel odor, flooring, and light, for cue dependent memory testing. Following a 2-min habituation, the tone was presented thrice for 30 s with an interval of 1 min in between each tone. Freezing during the three tone periods was recorded. The EthoVision XT 10- Noldus was used to analyze the videos.

### Social interaction test

The test took place in a Non-Glare Perspex box (60 × 40 cm) with two partitions that divide the box to three chambers, left, center and right (20 × 40 cm). The mouse is placed in the middle chamber for habituation (5 min) when the entry for both side chambers is barred. Test mouse was then allowed to explore the whole arena (10 min), where they freely choose between interacting with a novel mouse in one chamber or stay in an empty chamber. During this time, a camera films and tracks the behavior of the animals, including time spent in each chamber.

### Immunostaining

Mice were perfused with 4% paraformaldehyde and then brains were dissected and incubated in 30% sucrose for 2 days. 30 μm slices sections were taken by sliding microtome. Slices were blocked for 1 h in blocking solution (10% horse serum, 0.3%triton and 1XPBS, and then incubated with primary antibodies for PV (1:500, Sigma) or SST (1:100, Millipore) overnight at room temperature. The following day, slices were washed with incubated for 1 h with secondary antibodies (alexa488 and cy3), stained for 5 min with Hoechst (Sigma), and washed three times, followed by mounting.

For counting PV and SST neurons, four brain of 2 weeks and 11 weeks old mice were perfused each group (WT and CTCF cKO), and 3–4 slices were taken from different brain region, hippocampus and prefrontal cortex. After immunostaining, the number of PV and SST neurons was counted manually and calculated per ${\mathrm{mm}}^{2}$.

For checking KCNC1 florescence intensity in PV neurons, four brain of 2 weeks old mice were perfused each group (WT and CTCF cKO), and 3–4 slices were taken from different brain region, hippocampus and prefrontal cortex. After immunostaining, by using ZEN program, the results of KCNC1 florescence intensity were expressed as the mean fluorescence intensity in arbitrary units per PV cell.

### Tunel staining

30 μm thick floating sections of 4% paraformaldehyde fixed mice brain were used to analyze apoptotic cells. The brain slices were processed using the In Situ Cell Death Detection Kits (Roche Life Science) according to manufacturer's instruction.

### Single-nucleus extraction, RNA sequencing, and analysis

For the single-nucleus RNA-seq experiment, 2 weeks old mice were sacrificed. The forebrain of each mouse were dissected and minced using homogenizer in 5 ml homogenization buffer (0.25 M sucrose, 25 mM KCl, 5 mM MgCl2, 20 mM Tricine-KOH, 1 mM DTT, 0.15 mM spermine, 0.5 mM spermidine), 7 µl RNase Inhibitor (Promega N2611) and 300 µl 5% IGEPAL. The sample was filtered through a 40 µm strainer, mixed with 5 ml of 50% iodixanol (Sigma D1556), underlayed with a gradient of 30% and 40% iodixanol, and centrifuged at 4600 *g* for 1 h in a swinging bucket centrifuge at 4 °C. Nuclei were collected at the 30–40% interface. The nuclei was transferred to a 2 ml tube and centrifuged 10 min at 1000* g* and 4 °C. Cell pellet was resuspended in PBS 1× , 0.04% BSA. Libraries for single nuclei RNA-seq were prepared using the Chromium Next GEM Single Cell 3′ GEM, Library & Gel Bead Kit v3.1 as recommended by the manufacturer. Cell-RT mix was prepared to aim 10,000 nuclei per sample and applied to Chromium Controller for GEM generation and barcoding. Libraries were sequenced on a NextSeq 550 sequencing system to a depth of approximately 30,000–40,000 reads per cell. Raw sequencing reads were analyzed using 10× Genomics Cell Ranger version 4.0.0 pipeline. Following fastq generation, counting was performed using a custom “pre-mRNA” reference package (based on Mus musculus mm10 genome), listing each gene transcript locus as an exon, in order to count intronic reads. Cell loupe browser was used to perform differential gene analysis and to visualize data. Gene ontology analysis was performed using Toppgene.

### Electrophysiology

Mice were anesthetized with a lethal dose of ketamine/xylazine mixture and checked for any reflex by a toe-pinch to ensure proper anesthesia. Following, mice were perfused with ice-cold cutting solution containing (in mM): 110 sucrose, 60 NaCl, 3 KCl, 1.25 NaH_2_PO_4_, 28 NaHCO_3_, 0.5 CaCl_2_, 7 MgCl_2_ and 5 D-glucose. 300-μm-thick coronal sections of dorsal hippocampus were taken using a SMZ7000 vibratome (Campden Instruments) in ice-cold cutting solution. To allow recovery of the slices after sectioning, the slices were transferred to artificial CSF (aCSF) containing (in mM): 125 NaCl, 2.5 KCl, 1.25 NaH_2_PO_4_, 25 NaHCO_3_, 25 D-glucose, 2 CaCl_2_, and 1 MgCl_2_ at 34 °C for 30 min. All of the solutions were continuously bubbled with 95% O_2_/5% CO_2_. After initial recovery, the slices were left for 60 min at room temperature (RT) before transferring to the recording chamber for additional 30 min of acclimation at RT.

Whole-cell voltage-clamp recordings: Hippocampal CA1 pyramidal neurons were visualized using infrared differential interference contrast (IR-DIC) microscopy and identified by their morphology and firing pattern. All recordings were carried out at RT. For whole-cell recordings, (3–5 MΩ) borosilicate glass pipettes (1B150F-4, WPI inc.) were pulled (P-1000; Sutter Instruments, Navato, CA). For measuring inhibitory postsynaptic currents (IPSCs), the pipettes were back-filled with Cs-based intracellular solution containing (in mM): 140 CsCl, 1 EGTA, 6 KCl, 4 NaCl, 2 MgCl_2_, and 10 HEPES, pH 7.3 (adjusted with CsOH), and 290 mOsm, yielding a Chloride reversal potential of around 0 mV. Further, 6,7-dinitroquinoxaline-2,3-dione (DNQX) 40 µM and 2-amino-5-phosphonopentanoate (D-AP-5) 50 µM (Tocris, Ellisville, MI) were added to the aCSF to block glutamatergic AMPA and NMDA currents, respectively. Morphologically-identified pyramidal neurons were patched to reach a seal resistance of > 2 GΩ, and next the seal was ruptured into whole-cell mode. Following, we waited for at least 10 min for proper diffusion of the internal solution throughout the cell prior to the recordings. Recordings were performed in voltage clamp mode at − 70 mV for 60 s. Series resistance (R_s_) was monitored throughout each experiment and neurons with a series resistance R_s_ > 20 MΩ were excluded from analysis. The liquid junction potential was not corrected. Recordings were amplified using a Multiclamp 700B amplifier (Molecular Devices),  low-pass filtered at 2 kHz for all the volatge-clamp recordings, digitized by Digidata 1440 (Molecular Devices) and sampled at 20 kHz. All IPSC events were detected offline using the template search feature for event detection in ClampFit software (pClamp10, Molecular Devices). All events less than 7 pA in amplitude and their corresponding inter-event intervals were discarded from data analysis.

### Statistical analysis

Statistical analysis data were judged, and reported in figures and the figure legends, to be statistically significant when p < 0.05 by two-tailed t-test. Data are presented as mean ± SEM, and the number of animals (n) is mentioned.

## Results

### Characterization of CTCF- cKO in parvalbumin GABAergic interneurons

**Fig. 1:**
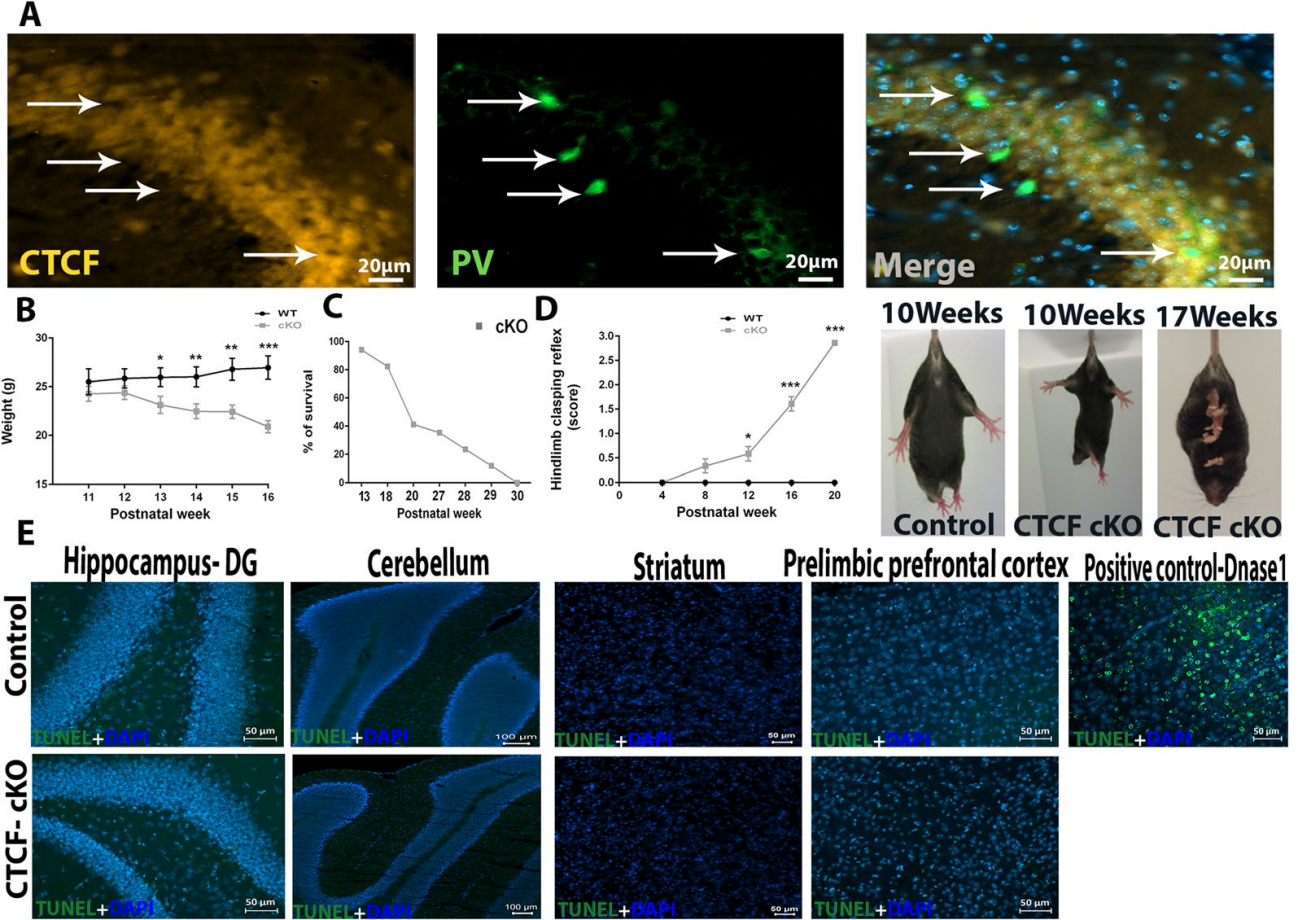
Characterization of the PV-Cre-Mediated CTCF cKO Mice. **A** Immunofluorescence analysis of CTCF in the hippocampus of 8-week-old wild-type and CTCF-cKO mice shows depletion of CTCF from parvalbumin inhibitory neurons. **B** Body weight over time. The CTCF-cKO mice exhibit significantly less weight by 13 weeks after birth. n = 7 for each group. *p = 0.016, **p = 0.005, ***p = 0.0006. Error bars represent SEM. **C** Survival curves of CTCF-cKO mice. 60% died by the time of 20 weeks after birth (n = 20). **D** 12 week old CTCF-cKO mice started to show an abnormal limb-clasping reflex when suspended by the tail which gradually got worse. WT = 5 animals, cKO = 12 animals. *p = 0.025, ***p = ${4.71}^{-6},$***p = ${4.4}^{-16}$. Error bars represent SEM. **E** Tunel analysis revealed no apoptosis in CTCF cKO mice in the hippocampus-DG, cerebellum, striatum and prelimbic prefrontal cortex of 17 week old mice compared to WT. Positive control was performed with Dnase1 treatment and the image taken on the prelimbic prefrontal cortex. n = 4 for each group

To investigate CTCF’s role in parvalbumin-expressing inhibitory neurons, we crossed CTCF^loxP^ mice with PV-Cre mice, which express Cre recombinase under the control of the promoter for PV, the gene encoding parvalbumin. The Cre-mediated deletion in CTCF-cKO mice was confirmed by immunohistochemistry analysis of the hippocampus (Fig. 1A). The CTCF-cKO mice display normal growth until postnatal week 13, where they begin to display decreased weight (Fig. 1B; *p = 0.016, **p = 0.005, ***p = 0.0006). By 17 weeks of age, the mice displayed a severe motor deficit phenotype, which was clearly visualized by disturbed gait as well as disturbed clasping (Fig. 1D; *p = 0.025, ***p = ${4.71}^{-6},$***p = ${4.4}^{-16}$), and 60% of mice died within 20 postnatal weeks (Fig. 1C). There was also a minor deficit in the limb clasping reflex as early as 12 weeks of age. To determine if cell death may be involved in these severe motor phenotypes, we performed tunel staining to identify apoptotic cells. No apoptosis was seen in the prelimbic prefrontal cortex, striatum, cerebellum and the hippocampus of CTCF- cKO mice (Fig. 1E, Additional file 1: Fig. S1). Due to the severe motor phenotype by 20 weeks of age, it is possible that the animal’s death is due to reduced ability to reach food and water.

### CTCF-cKO adult mice exhibit motor problems and reduction in anxiety-related behavior

**Fig. 2:**
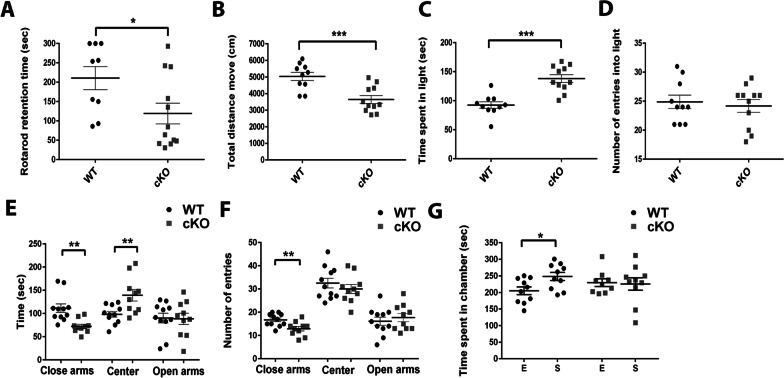
Adult CTCF-cKO mice demonstrate mild motor problems and reduction in anxiety-related behavior. Adult (8 weeks old) CTCF-cKO (n = 12) and WT (n = 9) performance in locomotor behavioral tests (**A**-**B**). **A** time spent on rotarod and **B** Distance move total. CTCF-cKO mice spent less time on the rotarod (*p = 0.035) and moved significantly less compared to the control group (***p = 0.00071). **C**, **D** Results of the light/dark transition test. CTCF cKO mice spent greater time in light zone (**C**, ***p = ${6.25}^{-5}$) however, similar number of entries into light zone (**D**). **E**, **F** Results of the elevated plus maze test. CTCF cKO mice spent less time in close arms (**p = 0.0014) however, more time in the center (**p = 0.0068) (**E**) and lower number of entries into close arm (**p = 0.0063) (**F**). **G** Results of the three-chambered social test. CTCF-cKO mice displayed no preference to spend time in a chamber with a stranger mouse (S) in comparison to empty chambers (**E**) (*p = 0.019). two-tailed, paired t test. Values in graphs are expressed as the mean ± SEM

To more precisely determine the phenotype induced by CTCF knockout in PV cells, we further evaluated adult male mice at least 8-weeks-old, before visually obvious motor impairment. CTCF-cKO mice spent less time on the rotarod (Fig. 2A; *p = 0.035) and moved significantly less (Fig. 2B; ***p = 0.00071) compared to the control group. These results indicate that CTCF-cKO mice already start developing motor deficits as early as 8 weeks of age.

Anxiety-related tests were then performed on the CTCF cKO mice at the same time period. In dark light test, CTCF-cKO mice spent more time in the light chamber at the dark light test (Fig. 2C; ***p = ${6.25}^{-5}$), without changes in the number of entries into the light zone (Fig. 2D). In addition, in the elevated plus maze test CTCF-cKO mice spent less time in the close arms and more time in the center zone (Fig. 2E; **p = 0.0014, **p = 0.0068 respectively). They also displayed a smaller number of entries into the close arms (Fig. 2F; **p = 0.0063). Therefore, at 2 months of age, the CTCF-cKO mice showed less anxiety-like behavior. Furthermore, we did social interaction test to test sociality. Unlike wild types, CTCF-cKO mice exhibit no preference to spend time in a chamber with a stranger mouse in comparison to empty chambers (Fig. 2G; *p = 0.019). These results indicate that at 8 weeks of age, behavioral phenotypes include mild motor impairment, decrease in anxiety-like behavior, and less sociability.

### CTCF-cKO four weeks old mice exhibit reduction in anxiety-related behavior with no motor problems

**Fig. 3:**
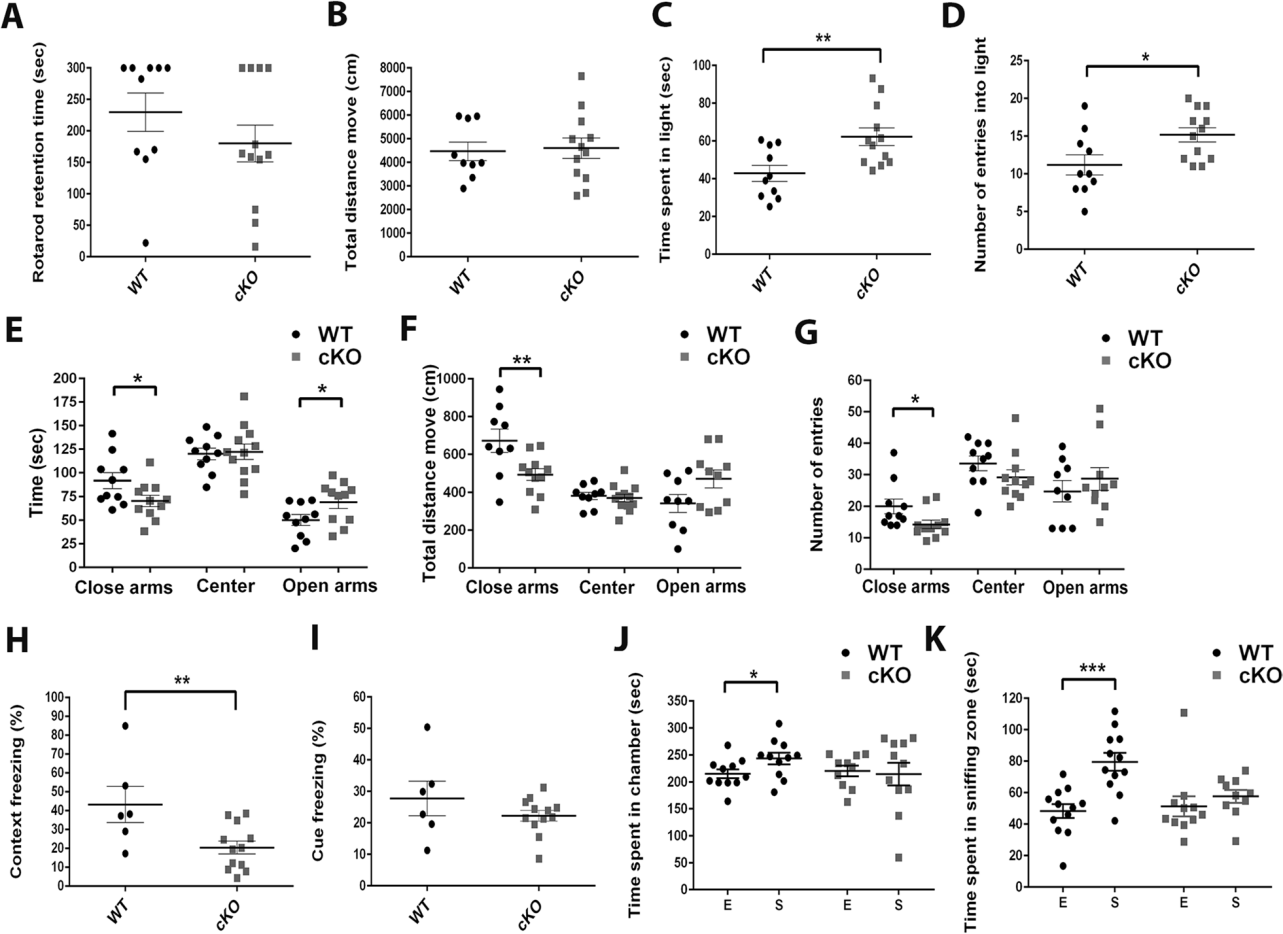
Four weeks old CTCF-cKO mice exhibit reduction in anxiety-related behavior with no motor problems. Four weeks old CTCF- cKO (n = 12) and WT (n = 10) (**A**, **B**) performance in locomotors behavioral tests. time spent on rotarod (**A**) and Distance move total (**B**), were not different between CTCF-cKO and WT mice. **C**, **D** Results of the light/dark transition test. CTCF cKO mice spent greater time in light zone (**p = 0.007) **C** and higher number of entries into light zone (*p = 0.022) (**D**). **E**–**G** Results of the elevated plus maze test. CTCF cKO mice spent less time in close arms (*p = 0.028) and more time in the open arms (*p = 0.044) (**E**), moving less in the close arm (**p = 0.002) (**F**) and had lower number of entries into close arm (*p = 0.043) (**G**). (**H**, **I**) CTCF-cKO mice showed less freezing 24 h after training in the contextual fear conditioning test (*p = 0.013) (**H**) and tendency of decline freezing during tones in the cue-dependent fear conditioning test (**I**) (WT n = 6, CTCF cKO n = 12). **J**, **K** Results of the three-chambered social test. CTCF-cKO mice displayed no preference to spend time in a chamber with a stranger mouse compered to control group (*p = 0.046) (**J**) and specific in the sniffing area (***p = 0.0002) (**K**) comparison to empty chambers. two-tailed, paired t test. Values in graphs are expressed as the mean ± SEM

To evaluate loss of CTCF expression in the PV inhibitory neurons on behavior at earlier developmental time point, we performed a battery of behavioral tests on CTCF-cko mice using 4 weeks old mice. CTCF-cKO mice and wild type mice spent the same amount of time on the rotating rotarod (Fig. 3A) and the distance that the mice travelled in the open field environment were equal (Fig. 3B). Therefore, 4 weeks old CTCF-cKO mice had no reduction in motor performance.

We further analyzed anxiety-like behavior. In dark light test CTCF-cKO mice spent significant more time in the light (Fig. 3C; **p = 0.007) and higher number of entries into the light chamber (Fig. 3D; *p = 0.022). In addition, in the elevated plus maze test CTCF-cKO mice spent less time in the close arms and more time in the open arms (Fig. 3E; *p = 0.028, *p = 0.044 respectively), traveled less distances in the close arms (Fig. 3F; **p = 0.002) and had a smaller number of entries into the close arms (Fig. 3G; *p = 0.043). Moreover, we tested behavior at the 4 week time point in the fear conditioning paradigm. In the contextual fear conditioning paradigm, CTCF-cKO mice exhibited decrease in freezing levels (Fig. 3H; *p = 0.013) while in cue test, CTCF-cKO mice displayed the same freezing levels compered to WT mice (Fig. 3I). Thus, three separate tests indicate that low expression of CTCF in PV inhibitory neurons leads to decline anxiety-related behavior at a developmental time point that is distinct from motor deficits.

Social interaction tests found that the CTCF-cKO mice did not prefer to spend time in a chamber with a stranger mouse. In addition, they showed no preference to be in the sniffing zone of the stranger mouse compered to WT mice (Fig. 3J, K; *p = 0.046, ***p = 0.0002 respectively). This data verifies that low expression of CTCF in PV inhibitory neurons decreases social behavior at a developmental time point that is distinct from motor deficits.

### CTCF-cKO mice display modified amounts of PV and SST neurons

**Fig. 4:**
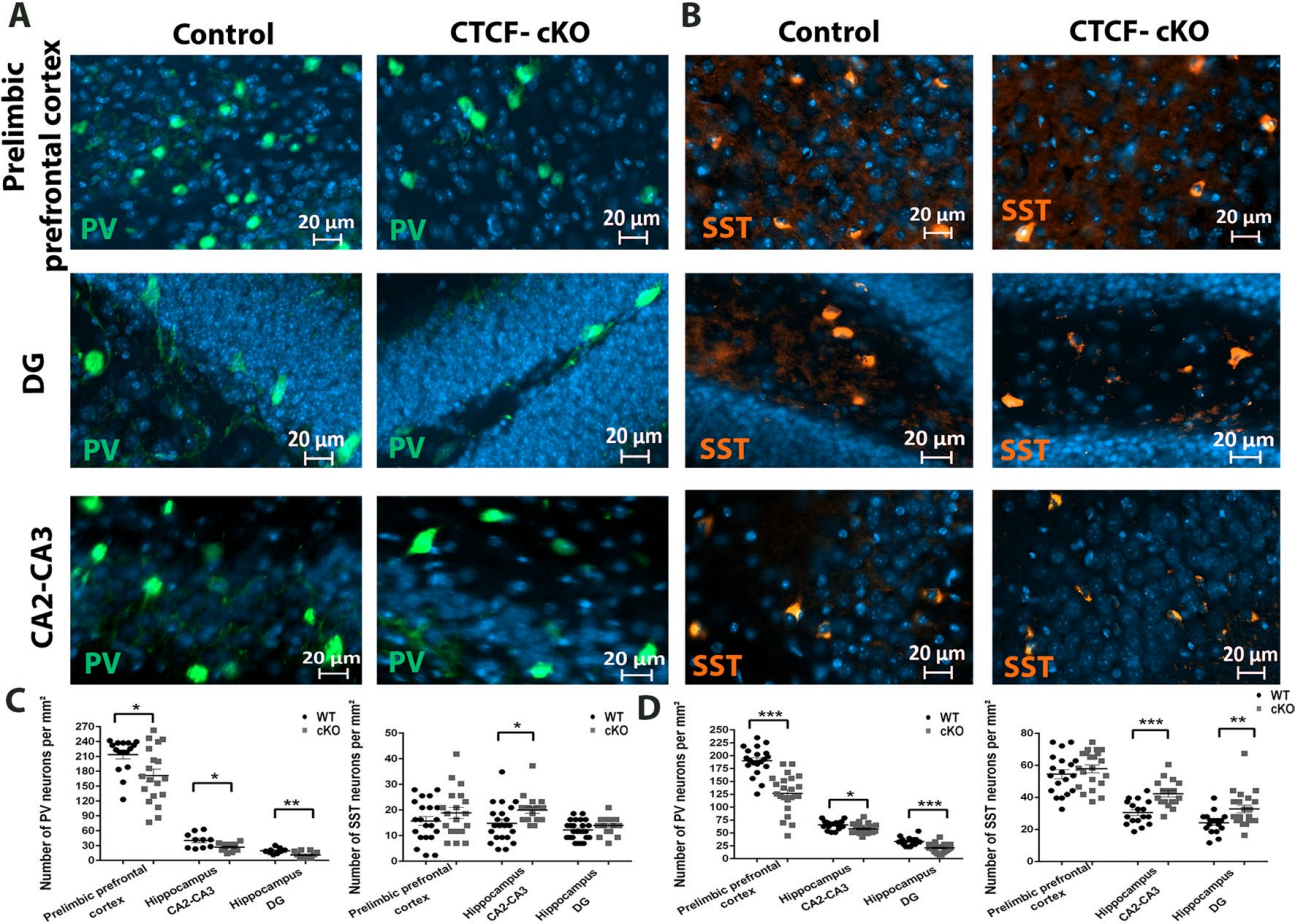
Dysregulation in the number of PV and SST neurons in CTCF-cKO mice. **A**, **C**, **D** CTCF-cKO mice exhibit decline in the number of PV neurons in the prelimbic prefrontal cortex and the hippocampal CA2-CA3 and DG of 11 weeks old (*p = 0.012, 0.015, **p = 0.009) (**C**) and 2 weeks old (***p = $${1.308}^{-7}, {2.43}^{-5}, *p=0.01)$$ (**D**) mice. Furthermore, (**B**, **C**, **D**) CTCF-cKO mice exhibit increase in the number of SST neurons particularly in the hippocampus of 11 weeks old (*p = 0.015) (**C**) and 2 weeks old (***p = 0.0001, **p = 0.007) (**D**) mice. However, no differences in SST neurons in the cortex of 2 and 11 weeks old mice. 4 animals per group; two-tailed t test. Values in graphs are expressed as the mean ± SEM

Approximately 70% of cortical interneurons can be divided into two practically non-overlapping groups based on their expression of parvalbumin or somatostatin [30–33]. It is well known that parvalbumin and somatostatin cortical interneurons originate in a subcortical embryonic structure called the medial ganglionic eminence (MGE). The fate of the PV and SST progenitors' cells depends on the transcription factors NKX2.1 and LHX6. Therefore, to understand the biological process which leads to CTCF-cKO phenotype, we counted the number of GABAergic neurons that express PV and SST in different brain regions. We found a decrease in PV + neurons in the cKO mice in the prelimbic prefrontal cortex and hippocampal CA2-CA3 and DG at both 11 weeks of age (Fig. 4A, C; *p = 0.012, *p = 0.015, **p = 0.009 respectively) and 2 weeks of age (Additional file 1: Fig. S2A, 4D; ***p=$${1.308}^{-7}, *\mathrm{p}=0.01, ***\mathrm{p}={2.43}^{-5}$$ respectively). Interestingly, we found increase in SST + neurons at 11 weeks of age (Fig. 4B, C; *p = 0.015) and 2 weeks of age in the hippocampus (Additional file 1: Fig. S2B, 4D; ***p = 0.0001, **p = 0.007). These findings suggest that CTCF have a role in the maintenance of neuronal identity.

### Single nuclei sequencing identifies gene expression changes involved in neuronal identity in CTCF-cKO mice

In order to more fully understand the possible roles for CTCF in neuronal subtype identity and gene transcription, we performed single-nuclei RNA-seq (snRNA-seq) from the 2 week mice forebrain both in wild type mice and CTCF cKO mice.

**Fig. 5:**
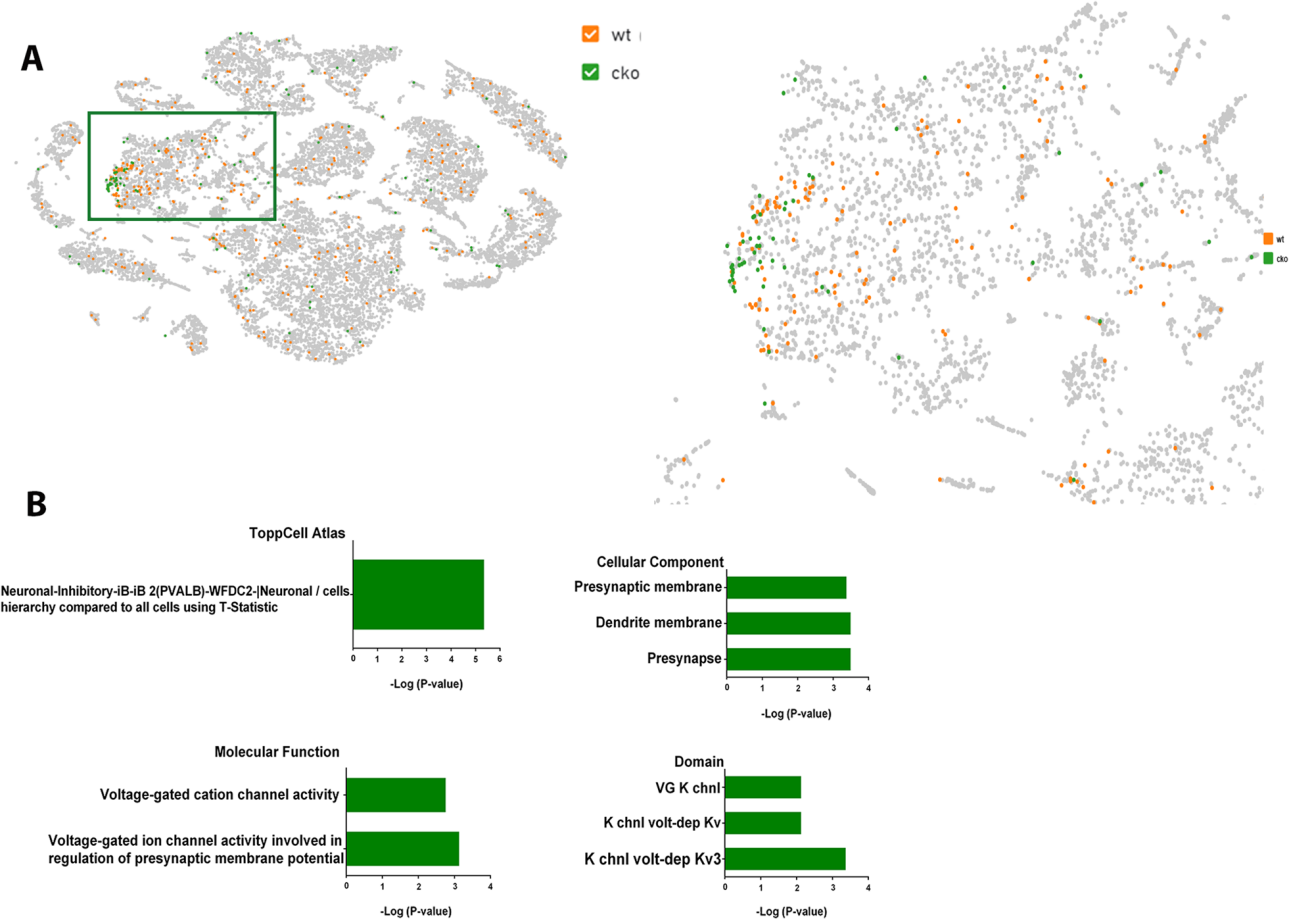
CTCF- cKO mice display decline in PV neurons and altered electrophysiological properties. **A**, **B** Results of the single-nuclei RNA-seq (snRNA-seq) from the 2 weeks mice forebrain. Picture on left shows all sequenced nuclei. Picture on right is zoom in on area with majority of parvalbumin-expressing neurons (represented by square on left side of panel). CTCF cKO mice are more densely clustered and less parvalbumin-expressing cells (**A**). Gene Ontology analysis of differentially expressed genes. Enrichment of upregulated genes highlighted ion channel activity, specifically potassium ion channels and genes that are involved in inhibitory neuron identity (**B**)

Approximately 1% of cells expressed parvalbumin. As expected from the immunohistochemistry data above, there was less parvalbumin-expressing cells in the CTCF cKO. Of interest, the CTCF cKO parvalbumin-expressing cells were more densely clustered, while the wild type parvalbumin-expressing cells revealed a more varied gene expression (Fig. 5A). We performed differential expression analysis specifically on the PV-expressing neurons between the two groups and found 18 genes that were differentially expressed in the CTCF cKO parvalbumin-expressing cells compared to wild type parvalbumin-expressing cells (Additional file 1: Table S1, FDR < 0.1). Gene ontology analysis revealed that CTCF cKO parvalbumin-expressing cells had a significant increase in genes that are involved in inhibitory neuron identity. In other words, these cells displayed a stronger transcriptional identity as inhibitory neurons. Gene ontology analysis also showed that these cells display an increase in genes involved in ion channel activity, particularly including genes Kcnc1 and Kcnc2, which are potassium ion channels critical for regulation of action potential. Overexpression of these genes have previously been shown to increase neuronal firing rate [34]. Therefore, the snRNA-seq data suggests that the remaining parvalbumin-expressing neurons in the CTCF cKO mice have a stronger identity as inhibitory neurons with altered electrophysiological properties (Fig. 5B).

### CTCF-cKO mice display increase in Kv3.1 in PV neurons and increased inhibitory currents at the pyramidal neurons in the hippocampus

**Fig. 6:**
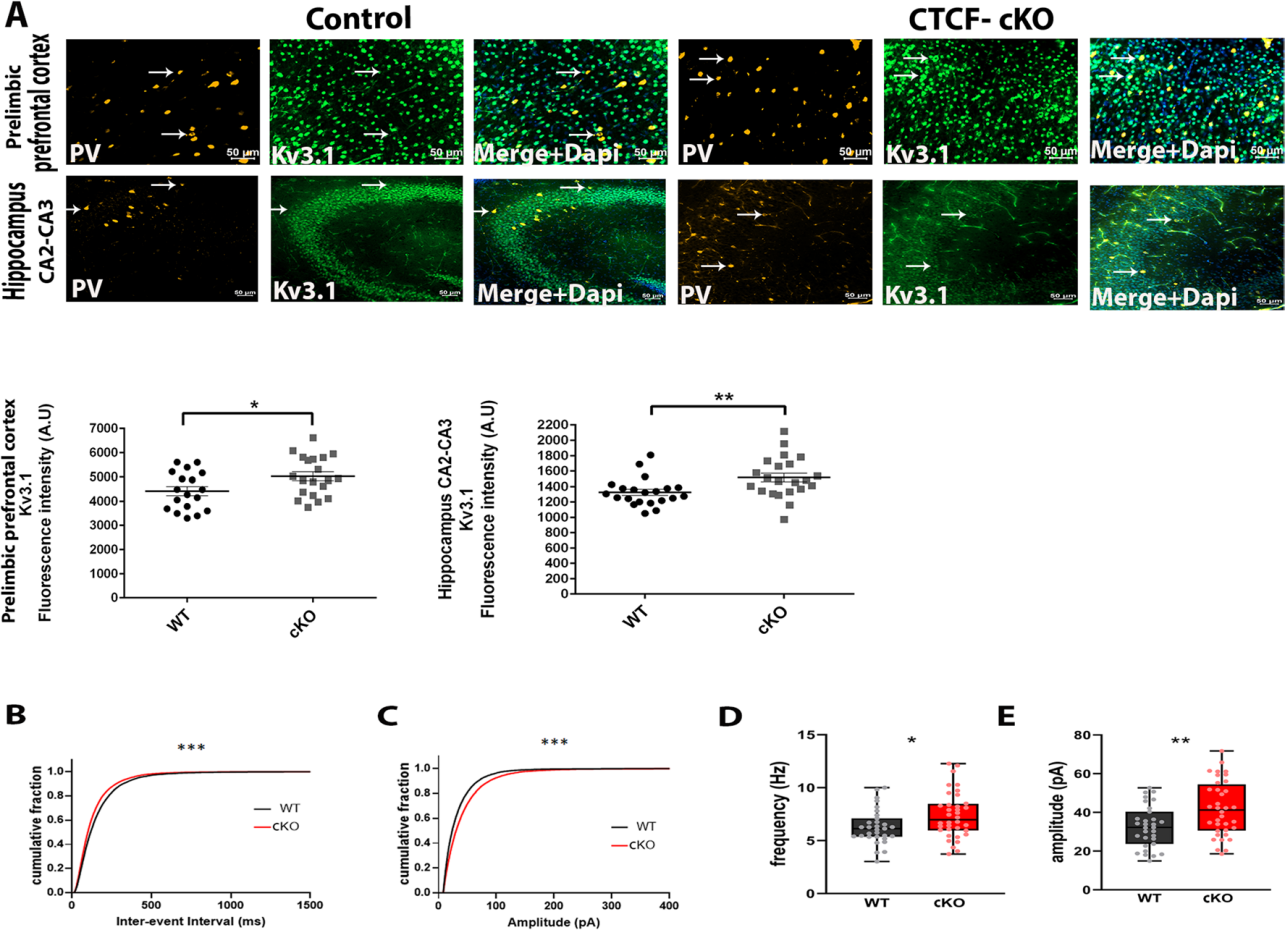
CTCF-cKO mice display increase in Kv3.1, potassium ion channel, in PV neurons and increased sIPSC frequency and amplitude in hippocampal pyramidal neurons. **A** CTCF- cKO mice exhibit elevation in the Kv3.1 florescence intensity in PV neurons in the prelimbic prefrontal cortex (*p = 0.047) and the hippocampal CA2-CA3 (**p = 0.008). **B**–**E** sIPSC recordings in the pyramidal neurons from acute hippocampal slices of 8 weeks old mice. **B** Cumulative probability plots reveal a leftward shift in the sIPSC inter-event intervals (Kolmogorov–Smirnov test, ****p < 0.0001) and a **C** rightward shift in the sIPSC amplitudes (Kolmogorov–Smirnov test, ****p < 0.0001) in the cKO mice compared to their WT littermates. **D** Average frequency (*p = 0.024) and **E** amplitude (**p = 0.004) are increased in the pyramidal neurons of the cKO mice when compared to their WT controls. WT, n = 31 neurons, 4 animals; cKO, n = 36 neurons, 4 animals. Values in graphs are expressed as the mean ± SEM

snRNA-seq from forebrain of 2 week CTCF-cKO mice determined upregulation of genes expressing in potassium ion channels, especially Kcnc1 and Kcnc2. We further attempted to verify this finding at the protein level with immunohistochemistry. We focused on the PV neurons and calculated the average of fluorescence intensity of Kv3.1. We found an enrichment of Kv3.1, potassium ion channel, in PV neurons in the cKO mice in the prelimbic prefrontal cortex and hippocampal CA2-CA3 at 2 weeks of age (Fig. 6A–C; *p = 0.047, **p = 0.008). To further determine how these changes in potassium channels in PV neurons affect the synaptic input to the pyramidal neurons, we studied the extrinsic properties by measuring the spontaneous inhibitory postsynaptic currents (sIPSCs) in the hippocampal CA1 pyramidal neurons using whole-cell voltage-clamp recordings. We found a leftward shift in the cumulative distribution curves of the interevent intervals (Fig. 6B; ****p < 0.0001), with a corresponding increase in the mean frequency of the IPSCs (Fig. 6D; *p = 0.024). Additionally, we observed a rightward shift in the cumulative distribution of amplitudes (Fig. 6C; ****p < 0.0001), with a corresponding increase in the mean IPSC amplitude (Fig. 6E; **p = 0.004). Taken together, we observe an enhanced inhibitory tone on the pyramidal neurons. Therefore, these data together suggest that depletion of CTCF augments the levels of potassium ion channels in PV neurons, which further modulate the inhibitory inputs to the pyramidal neurons of the hippocampus, either directly or via homeostatic network responses.

## Discussion

This study describes the effects of depleting CTCF in parvalbumin expressing neurons. We have demonstrated that mice with CTCF cKO in PV-expressing inhibitory neurons exhibit reduction in anxiety-related behavior, less sociability and a decline in motor performance over time. The anxiety-related behavior and sociability decline are seen before decline in motor performance, demonstrating temporal-specific effects.

Genetic studies discovered individuals with de novo mutations in CTCF. The clinical spectrum is highly variable [7–10], but often includes intellectual disability, microcephaly, and growth retardation in different severity levels. Behavioral abnormalities are common, including cases of temper tantrums and/or autism.

We have found that knockdown of CTCF in parvalbumin-expressing cells induces a decrease in sociability. As explained above, some patients with CTCF mutations are diagnosed with autism, which includes social deficits. There have been conflicting results in previous studies of CTCF knockdown in excitatory neurons whether that knockdown produces direct effects on sociability. Therefore, our results indicate that CTCF may regulate social behavior partially through effects in subsets of inhibitory neurons.

Some of the individuals with haploinsufficiency of CTCF observed motor problems, including delay in the age of walking and muscular hypotonia. Skeletal X-ray analysis showed "a diffuse osteopenic texture of the bones along with a brachycephalic type of craniosynostosis" [9]. Furthermore, all patients had feeding difficulty after birth. Considering the behavioral phenotype, motor dysfunction and weight loss of our CTCF- cKO mice, it is possible that these similarity in clinical features may partly be due to deficits in inhibitory neurons.

Previous studies have determined that CTCF is an important regulator of the expression of Pcdh genes, which are central in the mouse and human brain into specifying the identity and diversity of individual neurons [35–37]. In immunohistochemistry analysis, we found decrease in the number of PV neurons and increase in the number of SST neurons. Single nuclei RNA sequencing results also determined a role for CTCF in cell identity. These data confirm the essential of CTCF in neuronal diversity and specification. In single nuclei RNA-seq analysis, we found that deletion of CTCF in parvalbumin-expressing neurons induced specific changes in gene expression and neuronal identity that may help to explain the behavioral phenotypes. Parvalbumin-expressing cells displayed an increase in Kv3.1 potassium channel expression, which we further verified by immunohistochemistry in both prelimbic prefrontal cortex and hippocampus. Previous studies have shown that increase of these channels increases the firing frequency of parvalbumin-expressing cells [34]. Further studies found that increasing the firing frequency of these cells induces an anxiolytic effect and changes in sociability in mice [38]. Therefore, changes in potassium ion channels may partially explain the behavioral changes seen in our mice. In addition, gene ontology analysis found an increased expression of genes involved in inhibitory neurons identity in the CTCF cKO mice, further suggesting an increase in inhibitory neurons function.

In order to gain further insights into how cellular and gene expression changes may be affecting neuronal function and inhibitory drive, we performed electrophysiology on pyramidal neurons of the hippocampus. We found a higher sIPSC frequency and amplitude in the hippocampal pyramidal neurons of the cKO mice. There are two possible interpretations of these results. The Kv3.1 channels that are predominantly expressed in PV-positive interneurons, and absent in SST-positive interneurons of the hippocampus [39, 40], are increased in the PV-positive neurons of cKO mice. Therefore, this increase in Kv3.1 channels, which are responsible for the regulation of the action potential (AP) firing frequency by rapid repolarization [41], might lead to an increased global inhibitory input to the hippocampal CA1 in the cKO mice. The increased Kv3.1 channel expression can also be correlated with the reduced locomotion in the cKO mice, based on previous studies. Interestingly, a previous study found that knockout of Kv3.1/Kv3.3 potassium channels lead to hyperactivity [42]. Another possible interpretation is that the inhibitory drive on to the pyramidal neurons is not entirely dependent on the PV-positive interneurons but in part on SST-positive interneurons. PV and SST interneurons are two major inhibitory cell types found within the neocortex and hippocampus with axonal arborizations preferentially targeting the perisomatic and distal dendritic regions of the pyramidal neurons, respectively [43]. Correspondingly, we posit that this increase in the synaptic properties in the CA1 pyramidal neurons could be due to the observed elevated levels of SST interneurons in the hippocampus. Taken together, the gene expression and electrophysiological data suggest an increase in inhibitory drive which may be leading to the downstream behavioral and motor phenotypes.

Comparison of our findings with transgenic knockouts of CTCF-binding partners suggests specific roles for a CTCF complex in behavior. For example, mice with methyl-CpG binding protein 2 (MeCP2) cKO in inhibitory neurons developed motor and social deficits with no anxiety related behavior [44]. Other study on MeCP2 cKO in excitatory neurons during development induced heightened anxiety and abnormal social interactions [45]. Previous study on Smc3 cKO mice also exhibited more anxiety-related behavior [46].

A previous study induced CTCF depletion in precursors of cortical interneurons [27], which include both parvalbumin and somatostatin-expressing neurons. They found a marked decrease in cortical interneuron migration and numbers of both somatostatin and parvalbumin-expressing neurons. In contrast, in our parvalbumin-expressing cell CTCF knockout we find decrease of parvalbumin-expressing cells but increase of somatostatin-expressing cells. Since the knockout in the previous study was in an earlier developmental time point, this might explain a decrease in differentiation into both cell types. However, our knockout was induced at a later time point, after differentiation into parvalbumin-expressing cells. The increase of somatostatin-expressing cells in several brain regions may suggest a trans differentiation of parvalbumin-expressing cells to somatostatin-expressing cells after CTCF depletion.

Gene ontology analysis on PV neurons exhibited upregulation of genes that are involved in inhibitory neuron identity. Recent study on the lysine acetyltransferases type 3 (KAT3) family members CBP and p300 that are important transcriptional co-activators show this genes importance in cell type-specific genes maintains and neuronal identity [47]. Therefore, different epigenetic regulators may have specific roles in regulating neuronal identity of specific cell types.

In summary, our study provides novel data on the function of CTCF in PV inhibitory neurons in brain. These results suggested that CTCF in GABAergic inhibitory PV neurons is essential for mice motor function, anxiety, and sociability like behavior together with the involvement of CTCF in neuronal identity.

## Supplementary Information

**Additional file 1: Table S1.** Differentially expressed genes in parvalbumin-expressing cells between wild type and CTCF-cKO mice. **Figure S1.** No apoptosis seen in hippocampus of CTCF cKO mice. **Figure S2.** Dysregulation in the number of PV and SST neurons in two weeks old CTCF- cKO mice.

## Data Availability

The datasets used and/or analyzed during the current study are available from the corresponding author on reasonable request.
