# Supplementary material for: CTCF in parvalbumin-expressing neurons regulates motor, anxiety and social behavior and neuronal identity
Source: Mol Brain. 2022 Apr 4;15:30. doi: 10.1186/s13041-022-00916-9 (PMC8981645; doi:10.1186/s13041-022-00916-9)
Supplement: Supplementary file 1 — Additional file 1: Table S1. Differentially expressed genes in parvalbumin-expressing cells between wild type and CTCF-cKO mice. Figure S1. No apoptosis seen in hippocampus of CTCF cKO mice. Figure S2. Dysregulation in the number of PV and SST neurons in two weeks old CTCF- cKO mice. [file 13041_2022_916_MOESM1_ESM.docx]

**List of Contents**

Table S1: Differentially expressed genes in parvalbumin-expressing cells between wild type and CTCF-cKO mice

Figure S1: No apoptosis seen in hippocampus of CTCF cKO mice

Figure S2: Dysregulation in the number of PV and SST neurons in two weeks old CTCF- cKO mice

**Table S1: Differentially expressed genes in parvalbumin-expressing cells between wild type and CTCF-cKO mice**


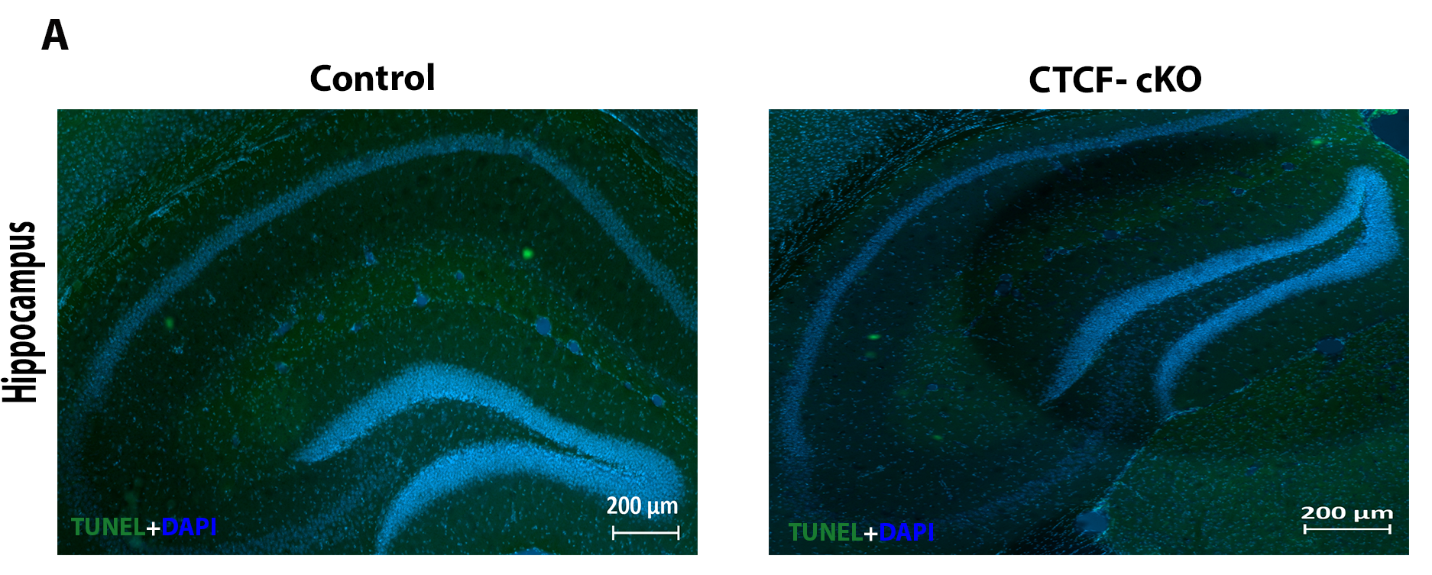


**Figure S1: no cell apoptosis was seen in the hippocampus of CTCF cKO mice.** (A) Tunel analysis indicate no apoptosis in CTCF-cKO or wild type hippocampus.


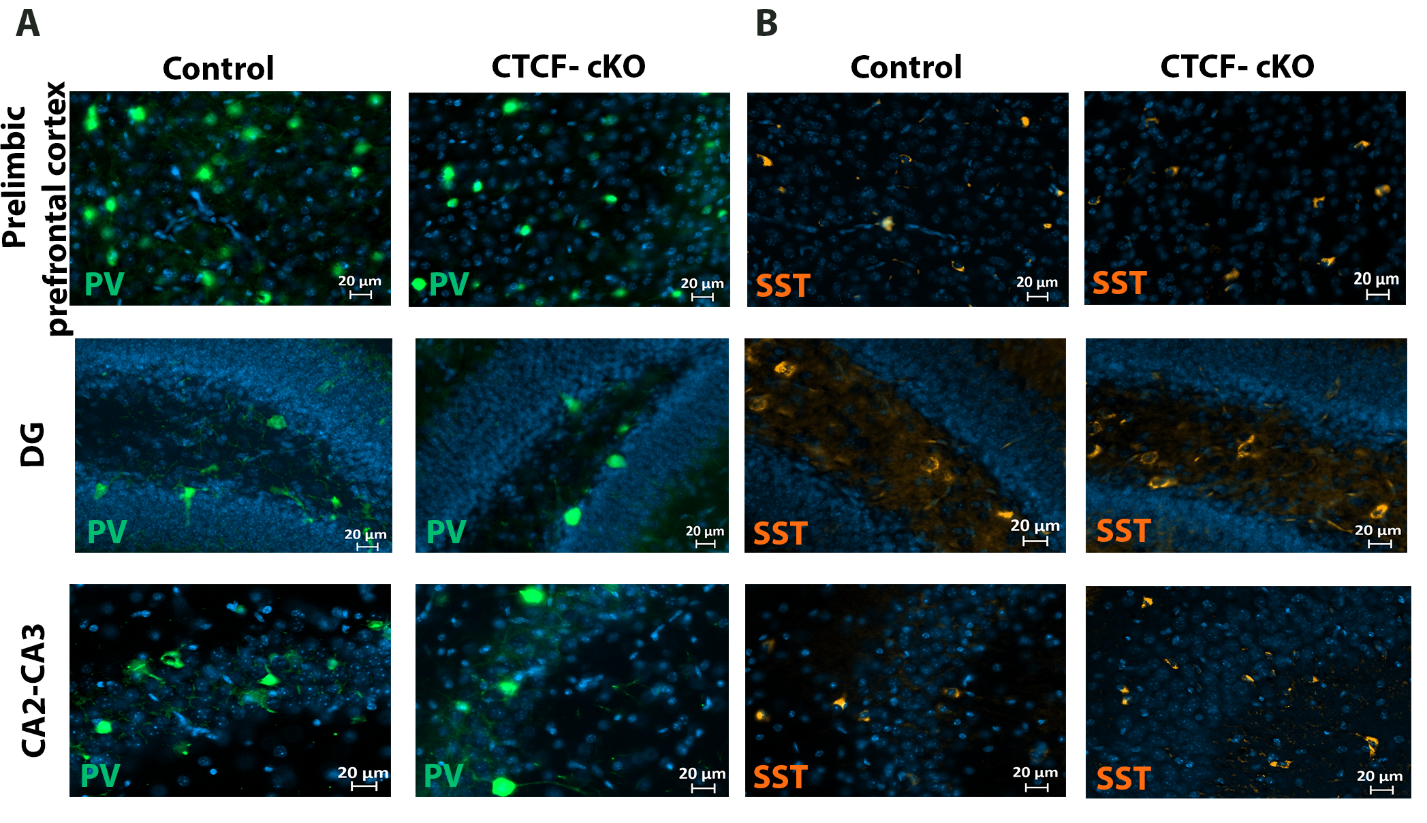


**Figure S2: Dysregulation in the number of PV and SST neurons in two weeks old CTCF- cKO mice.** (A) CTCF- cKO mice exhibit decline in the number of PV neurons in the prelimbic prefrontal cortex and the hippocampal CA2-CA3 and DG of two weeks old CTCF- cKO mice. (B) CTCF- cKO mice had incline in the number of SST neurons in the hippocampal CA2-CA3 and DG of two weeks old CTCF- cKO mice, with no differences in the prelimbic prefrontal cortex. The plots of this data can be found in Figure 4D of the main manuscript.
